# In Vitro Evaluation of Antimicrobial Synergy Against Multidrug-Resistant Gram-Negative Paediatric Bloodstream Pathogens in South Africa

**DOI:** 10.3390/antibiotics14070630

**Published:** 2025-06-20

**Authors:** Prenika Jaglal, Sithembiso Christopher Velaphi, Colin Nigel Menezes, Khine Swe Swe-Han

**Affiliations:** 1 Department of Clinical Microbiology and Infectious Diseases, National Health Laboratory Service and Faculty of Health Sciences, University of Witwatersrand, Johannesburg 2050, South Africa; 2 Department of Paediatrics and Child Health, Chris Hani Baragwanath Academic Hospital, School of Clinical Medicine, Faculty of Health Sciences, University of the Witwatersrand, Johannesburg 2050, South Africa; 3 Division of Infectious Diseases, Department of Internal Medicine, University of the Witwatersrand, Johannesburg 2050, South Africa; 4 Department of Medical Microbiology, Inkosi Albert Luthuli Central Hospital, National Health Laboratory Service, University of KwaZulu-Natal, Durban 4000, South Africa

**Keywords:** synergy testing, tigecycline, meropenem, carbapenem-resistant, Enterobacterales, *Acinetobacter* species, multidrug-resistant

## Abstract

**Background**: In vitro synergy testing (ST) is a useful means to gauge the performance ofantibiotic combinations against multidrug-resistant (MDR) Gram-negative bacteria (GNB). This study aimed to determine synergy of antibiotics against paediatric bloodstream (BS) carbapenem-resistant Enterobacterales (CRE) and extremely drug-resistant (XDR) *Acinetobacter* species. **Methods**: This cross-sectional study was conducted at a public tertiary hospital in South Africa, from January 2023 to December 2023. Sixty-eight isolates from children with bloodstream infections (BSI), comprising 55.9% (38/68) CRE and 44.1% (30/68) XDR *Acinetobacter* species, were performed ST using the fixed-ratio Epsilometer-test method. Combinations of colistin and meropenem, colistin and fosfomycin, colistin and tigecycline, meropenem and fosfomycin, meropenem and tigecycline, and fosfomycin and tigecycline were tested. **Results**: In vitro synergy for CRE was best demonstrated with tigecycline and meropenem, at 92.1% (35/38), and fosfomycin and meropenem at 73.7% (28/38). Among the XDR *Acinetobacter* species, the highest rates of synergy of 76.7% (23/30) were observed with tigecycline and meropenem. The absence of synergy was noted with colistin and meropenem for the CRE, with many displaying indifference and antagonism at rates of 65.8% and 22%. Most XDR *Acinetobacter* species (56.7%; 17/30) expressed indifference to colistin and meropenem with synergy and antagonism displayed in 23.3% and 10% of isolates. **Conclusions**: This study highlights tigecycline and meropenem displaying impressive in vitro synergy when compared to the in-use colistin and meropenem for CRE and XDR *Acinetobacter* species. Tigecycline and meropenem may be a viable salvage therapeutic option for MDR Gram-negative paediatric infections. Future research is warranted to confirm in vivo synergy clinically.

## 1. Introduction

The dreaded surge of global paediatric infections attributed to multidrug-resistant (MDR) organisms has resulted in nearly 700,000 deaths, with a third comprising the neonatal population [1]. MDR Gram-negative (GN) organisms such as *Acinetobacter* species (spp.), *Pseudomonas aeruginosa*, *Escherichia coli* and *Klebsiella pneumoniae* have contributed to the infectious burden in Europe, with alarming rates of carbapenem-resistant Gram-negative bacteria (GNB) reported from developing countries [2]. One of the primary challenges of antimicrobial resistance (AMR) is the limited availability of effective treatment options for MDR bacterial infections. The lack of paediatric pharmacokinetic and safety data for antimicrobials with the restricted approval of antibiotics in patients under 18 years old limits treatment options for infections caused by carbapenem-resistant organisms (CROs) in children. Studies conducted in adults have evaluated the combination of classic antibiotics with different mechanisms of action (e.g., polymyxins, fosfomycin, carbapenems) to facilitate synergy for treating infections caused by GN CROs [2,3].

Antibiotic synergy testing (ST) is a useful in vitro susceptibility method to determine whether the combination of antimicrobial agents is more active compared to the most active drug alone [4]. There are four primary methods that describe how synergy can be assessed in vitro, i.e., time-kill assays (TKA), checkerboard assays (CA), Epsilometer (E)-test methods, and the multiple combination bactericidal test [4,5]. Despite numerous ST strategies, there is no in vitro gold standard method, and inconsistent results between ST techniques have been cited as a concern [6].

The E-test is a gradient ST method employing the use of plastic strips coated with varying antimicrobial concentrations. To assess synergy, two strips of different agents are used to determine the fractional inhibitory concentration (FIC) index, which is the sum of the FICs of each drug tested when used in combination. The FIC for each drug is determined by dividing each drug’s (minimum inhibitory concentration) MIC when used in combination by each drug’s MIC when used alone [3,7]. Studies have reported the performance agreement for the E-test when compared to the TKA as 63–75% and the CA as 44–88% [7]. The relative simplicity of the E-test method has made it a worthy alternative to the CA and TKA for ST. The E-test method showed a better correlation with the TKA concordance of 80.6%, respectively [3].

Carbapenem-resistant Enterobacterales (CRE) and extremely drug-resistant (XDR) *Acinetobacter* spp. are a menace amongst paediatric units worldwide resulting in high fatality rates [8,9,10] ST can guide treatment decisions for MDR GNB especially when conventional testing does not reveal susceptibility to two agents from different antibiotic classes. Global studies performed in older children have successfully used tigecycline, despite a lack of paediatric safety/efficacy data, or fosfomycin in combination with either colistin, a carbapenem or an aminoglycoside [8,11,12]. Multitudes of reports have described in vitro synergy with colistin and meropenem, which has been the mainstay of treating CRE and the XDR *Acinetobacter* spp. in some paediatric units, including the study site [13]. In low and middle-income countries (LMIC) where MDR GN infections are prevalent, there remains a dearth of information regarding in vitro synergistic antibiotic activity targeting MDR GN causing infections in a paediatric population. This study aimed to determine the in vitro synergy of in-use antimicrobials colistin and meropenem, as well as fosfomycin (an old revived antibacterial agent) and tigecycline in combination, as potential treatment modalities for common MDR GN (CRE and XDR *Acinetobacter* spp.) causing bloodstream (BS) infection in paediatric units.

## 2. Results

### 2.1. MIC Distribution and Disk Diffusion Susceptibility Testing of Study Isolates

#### 2.1.1. Types of MDR GN Isolates Studied

A total of 68 MDR GN isolates from paediatric patients with BS infections (BSI) were tested comprising 38 (55.9%) CRE and 30 (44.1%) *Acinetobacter* spp. The carbapenemase types of CREs identified were oxicillinase (OXA-48) (*n* = 30; 78.9%) and New Delhi metallo-β-lactamase (NDM) (*n* = 8; 21.1%). The CRE studied was mainly *Klebsiella pneumoniae* (*n* = 35; 92.1%) and the remaining (*n* = 3; 7.9%) were *E. coli*.

#### 2.1.2. Susceptibility of MDR GN Isolates Against Individual Antibiotics

Among the CRE, 23.7% (9/38) had meropenem MICs of ≤1 µg/mL denoting susceptibility (8 *K. pneumoniae* and 1 *E. coli*), while the majority (77.1%; 27/35) of the carbapenem-resistant (CR) *K. pneumoniae* (CRKP) had resistant MICs of ≥2 µg/mL (Table 1). According to the Kirby Bauer disk diffusion (KBDD) method, only three CRKP isolates were susceptible to aminoglycosides (gentamicin and amikacin) while two other isolates were susceptible to ciprofloxacin. All CRE were resistant to ampicillin, co-amoxiclav, ceftriaxone, piperacillin-tazobactam and cefepime. The majority (81.6%; 31/38) of CRE had tigecycline MICs of ≤2 µg/mL and were considered susceptible according to Clinical Laboratory Standards Institute (CLSI) and European Committee on Antimicrobial Susceptibility Testing (EUCAST) (Table 1) [14,15]. Colistin MICs were ≤2 µg/mL (intermediate range) for CRE, with none being colistin-resistant [14]. Although no fosfomycin clinical breakpoints exist, 50% of the CREs (19/38) had MICs ≤ 32 µg/mL, which are regarded informally as susceptible in the literature [16].

All XDR *Acinetobacter* spp. had meropenem MICs of 32 µg/mL, making them carbapenem-resistant *Acinetobacter* spp. (CRAS) with additional resistance noted to aminoglycosides, ciprofloxacin, ceftazidime, piperacillin-tazobactam and cefepime (KBDD). The vast majority of XDR *Acinetobacter* spp. isolates (29/30; 96.7%) exhibited tigecycline MICs ≤ 2 µg/mL, falling within the susceptible range. All XDR *Acinetobacter* spp. had colistin MICs within the intermediate range (≤2 µg/mL). CLSI colistin susceptible breakpoints do not exist for *Acinetobacter* spp. or Enterobacterales [14,15]. The majority of XDR *Acinetobacter* spp. (73.3%; 22/30) had fosfomycin MICs of ≥512 µg/mL (Table 2).

#### 2.1.3. Comparison of Colistin and Meropenem

**Figure 1:**
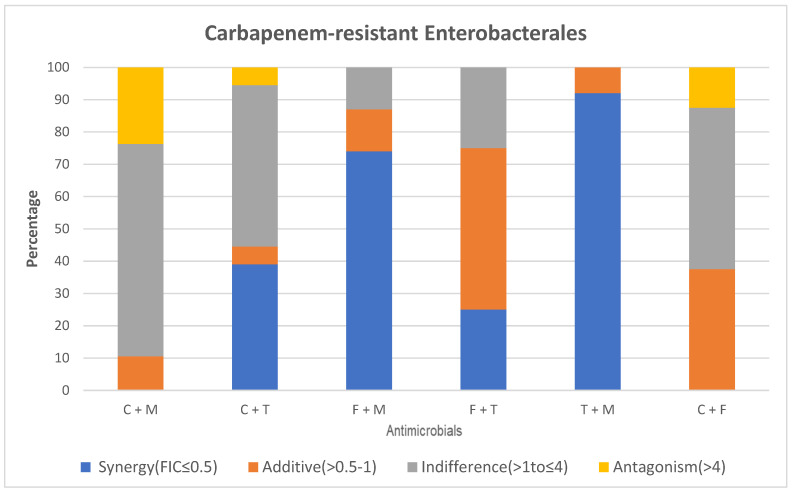
E-test synergy testing results for CRE. Key: FIC; fractional inhibitory concentration, C; colistin, T; tigecycline, M; meropenem, F; Fosfomycin.

Among the CRE, there was absence of synergy when colistin was tested with meropenem. The majority of CRE (65.8%) displayed indifference with 23.7% and 10.5% exhibiting an antagonistic and additive relationship (Figure 1).

**Figure 2:**
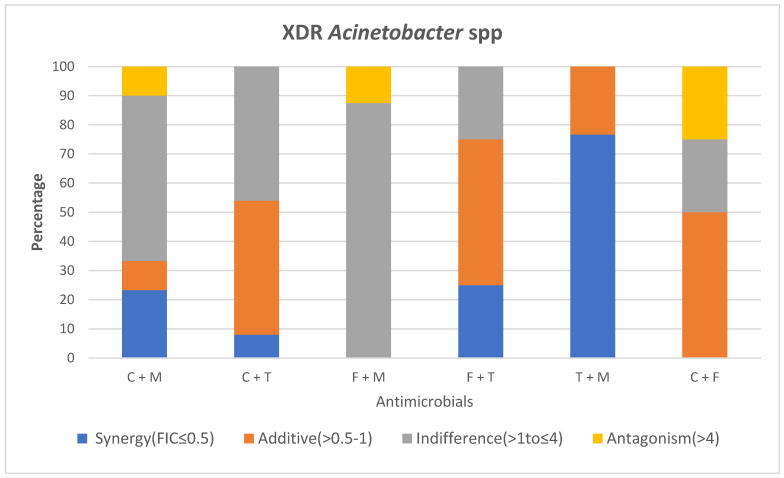
E-test synergy testing results for XDR *Acinetobacter* spp. Key: FIC; fractional inhibitory concentration, C; colistin, T; tigecycline, M; meropenem, F; Fosfomycin.

Colistin and meropenem displayed a synergistic effect against 23.3% of *A. baumannii*, with >50% displaying indifference. Ten percent were either antagonistic or additive (Figure 2).

#### 2.1.4. Comparison of Colistin and Tigecycline

The combination of colistin and tigecycline showed a synergistic effect against 39% of CRE while 50% expressed indifference, respectively.

*A. baumannii* revealed synergy in 8% of isolates, with the remaining being either additive (46%) or indifferent (46%).

#### 2.1.5. Comparison of Fosfomycin and Colistin

No synergy was observed when colistin was tested with fosfomycin for both the CRE and *A. baumannii*. Approximately 50% of CRE isolates displayed indifference, while 12.5% were antagonistic, and 37.5% were additive to this combination.

Fifty percent of the *A. baumannii* were additive to this antimicrobial combination with 25% each being either indifferent or antagonistic.

#### 2.1.6. Comparison of Tigecycline and Fosfomycin

Both CRE and *A. baumannii* showed synergistic and indifference at 25%, with an additive effect at 50%. No antagonism was observed in test isolates.

#### 2.1.7. Comparison of Tigecycline and Meropenem

The combination of tigecycline and meropenem showed a synergistic effect against 92% of CRE isolates, with 8% being additive and none displaying indifference or antagonism, respectively (Figure 1).

*A. baumannii* isolates displayed a synergistic effect at a rate of 76.7% with 23.3% exhibiting an additive effect (Figure 2).

#### 2.1.8. Comparison of Fosfomycin and Meropenem

The combination of fosfomycin and meropenem showed a synergistic effect against 74% of CRE isolates with 13% being either additive or indifferent, respectively. There was no antagonism observed.

Amongst *A. baumannii*, most isolates (87.5%) displayed indifference, while 12.5% were antagonistic. Neither synergy nor an additive effect was noted.

## 3. Ethical Considerations

Hospital approval was given by the hospital protocol review committee and management. The National Health Laboratory Service (NHLS) granted permission to conduct testing on bacterial isolates. All data, with unique patient identifiers, were stored in a password-protected document. Ethics approval to conduct the study was obtained from the University of the Witwatersrand Human Research Ethics Committee, clearance certificate number M220718.

## 4. Discussion

This single-site study revealed that the best synergy amongst the CRE isolates was displayed with tigecycline and meropenem at 92%. This was closely followed by fosfomycin and meropenem synergy at 74%, colistin and tigecycline at 39%, as well as fosfomycin and tigecycline at 25% among the CRE isolates. Absence of synergy was observed with colistin and meropenem among all CRE with majority displaying in vitro indifference (65.8%; 25/38). XDR *Acinetobacter* spp. expressed the greatest synergy with tigecycline and meropenem at 76.7%, followed by fosfomycin and tigecycline at 25%, as well as colistin and meropenem at 23.3%. Of clinical concern was the antagonistic relationship of colistin and meropenem for the CRE (23.7%; 9/38) and XDR *Acinetobacter* spp. (10%; 3/30) which is the current therapeutic regimen prescribed for these drug-resistant isolates at the study site.

The rise in AMR worldwide has triggered the adoption of the use of combination therapy, wherein one antimicrobial synergizes the efficacy of the other. In vitro synergy has been demonstrated with colistin and meropenem in the literature, which is the standard treatment option for CRE and XDR *Acinetobacter* spp. BS infections at the study site [16,17]. Synergism is derived from colistin targeting the lipopolysaccharide of the GNB disrupting the outer membrane while carbapenems inhibit cell wall synthesis by binding to penicillin binding proteins [18]. In recent decades, colistin has been revived due to the increase in MDR GNBs with few licensed antimicrobials for treatment, especially in the paediatric population [19]. Concerns about colistin toxicity and dosage optimization still exist [13]. Colistin use as part of combination therapy for resistant GNBs has shown merits in terms of improved survival and microbiological eradication, especially when used with a carbapenem, (MICs of 16–32 µg/L) at high doses via extended infusion [20].

Our test isolates comprised of predominantly CRKP (51.5%; 35/68) OXA-48 carbapenemase producers displaying multidrug-resistance to third/fourth generation cephalosporins, aminoglycosides and fluoroquinolones compelling use of combination broad-spectrum antimicrobials. ST failed to demonstrate synergy with colistin and meropenem among our study CRE isolates, however, other studies reported conflicting findings with notable synergy expressed using CA (88%) and E-test fixed ratio methods (82%) [21]. These discordant results could be due to the bacterial strain differences as well as diversity in ST methodologies [6]. Previous studies have highlighted synergy with colistin and a carbapenem among *Acinetobacter* spp. reporting the superiority of this combination with the possible addition of a third antimicrobial agent to further improve antibacterial activity [21]. Our study, however, found that colistin and meropenem showed poor in vitro synergy among XDR *Acinetobacter* spp. (23.3%; 7/30) with most isolates displaying indifference (65.8% CRE; 56.7% XDR *Acinetobacter* spp.).

Tigecycline works by binding to the 30S sub-unit of the bacterial ribosome, thus inhibiting protein synthesis [22]. A meta-analysis of randomized trials showed tigecycline monotherapy was associated with an increased risk of mortality when compared with other regimens. Its use has therefore been proposed as part of a combination regimen when other options for treating MDR bacterial infections are exhausted [23]. Laboratory studies performing TKA and CA have demonstrated in vitro synergistic activity of tigecycline when combined with aminoglycosides, colistin, levofloxacin and imipenem, which proved to reduce emergence of tigecycline resistance [16,24]. Our study highlighted synergy with tigecycline and meropenem among majority of the CREs (92%) composed of 97.2% *Klebsiella pneumoniae* (all OXA-48 producers) and 2.8% *E. coli* (NDM producers). Despite XDR *Acinetobacter* spp. displaying meropenem MICs of 32 µg/mL and all having tigecycline (MICs ≤ 2 µg/mL) within the susceptible range, 76.7% of the isolates showed synergy with this antimicrobial combination. Tigecycline is not approved for paediatric use due to a lack of safety and efficacy data; however, it may have a viable role as salvage therapy [25]. Studies performed amongst older children have used tigecycline in combination with either colistin, a carbapenem or an aminoglycoside [26].

Fosfomycin is another agent that has been used as a last resort option for CREs. The mechanism of action of this drug involves irreversibly inhibiting an early stage of bacterial cell wall biosynthesis [12,27]. This agent achieves adequate concentrations in plasma, urine, and cerebrospinal fluid (CSF) when used intravenously and retains antibacterial activity against the majority of CRE isolates, however, *Acinetobacter* spp. are intrinsically resistant to this antimicrobial agent [28]. Fosfomycin resistance occurs rapidly when used as monotherapy therefore combination use is encouraged [12]. TKAs have confirmed the synergistic bactericidal effects in *Acinetobacter* spp. using fosfomycin in combination with either amikacin, gentamicin, tobramycin, minocycline, tigecycline, or colistin, with more than 99.9% reduction in bacterial cells [28]. Despite Acinetobacter efflux pump-related intrinsic resistance to fosfomycin, we observed a synergistic effect of fosfomycin with tigecycline in 23.3% of the XDR *Acinetobacter* spp. CRE isolates expressed impressive synergy with fosfomycin and meropenem (93.3%; 28/30) similar to other studies using various ST methods [29].

Limitations of this study included the small sample size and isolates being of only one specimen type, as many test isolates became non-viable or were discarded in error before ST. Carbapenem-resistant *Klebsiella pneumoniae* and carbapenem-resistant *Escherichia coli* were the only Enterobacterales assessed due to their predominance in the paediatric unit during the study period. This study is the first of its kind conducted locally and may be used to benchmark further in vitro and in vivo studies regarding combination treatment strategies in order to update treatment protocols.

## 5. Materials and Methods

### 5.1. Study Design and Population

This was a descriptive, cross-sectional laboratory-based study conducted on bacterial isolates cultured from the blood of patients (≤14 years old) admitted to paediatric wards at Chris Hani Baragwanath Academic Hospital (CHBAH), in Johannesburg, South Africa from January 2023 to December 2023. We studied 68 bacterial isolates comprising 38 CRE (*Klebsiella pneumoniae* (*n* = 35), *Escherichia coli* (*n* = 3)) and 30 XDR *Acinetobacter* spp. that were convenience-based sample isolates.

### 5.2. Study Setting

#### 5.2.1. Laboratory Processing of Blood Culture Specimens

Paediatric admissions with provisional diagnosis of sepsis have blood cultures (BCs) sent to the National Health Laboratory Services (NHLS), an on-site microbiology laboratory, as part of the work-up for sepsis. These specimens are incubated in an automated continuous monitoring system (BacT/Alert system^®^, bioMerieux, Marcy l’Etoile, France) to detect organism growth/positivity based on a calorimetric principle. Positive BCs are processed according to laboratory standard operating procedures (SOPs) twenty-four hours a day. Gram-staining followed by overnight culture and bacterial identification is performed using the Vitek 2 automated system^®^ (bioMérieux) and API^®^ (bioMérieux). Antimicrobial susceptibility testing (AST) is performed and interpreted according to the CLSI M100 as per laboratory SOP using KBDD methodology [14]. The following antibiotic disks (MASTDISCS^®^ AST) are used: ampicillin (10 µg), amoxicillin/clavulanate (20/10 µg), ceftriaxone (30 µg), ceftazidime (30 µg), cefepime (30 µg) gentamicin (10 µg), amikacin (30 µg), ciprofloxacin (5 µg), piperacillin-tazobactam (100/10 µg), ertapenem (10 µg) meropenem (10 µg), and imipenem (10 µg). All CRE cultures have carbapenem (ertapenem, meropenem and imipenem) MIC determination using E-test gradient method.

All Enterobacterales that are resistant or intermediate to any of the carbapenems (ertapenem, imipenem and meropenem) or demonstrate carbapenemase production are reported as CRE. *Acinetobacter* spp. that display susceptibility to antimicrobials in two or less drug classes are termed XDR [30]. Preliminary and final microbiology results are communicated daily with clinicians allowing for appropriateantimicrobial therapy and applicable contact-based transmission precautions to be instituted timeously.

#### 5.2.2. Carbapenemase Determination in CRE

Phenotypic carbapenemase detection for CRE is performed as per laboratory SOP using the RESIST-5 O.K.N.V.I (CORIS BioConcept, Gembloux, Belgium) immunochromatographic lateral flow assay. This technology allows for the rapid detection of OXA-48 and its variants, NDM, *Klebsiella pneumoniae* carbapenemase (KPC), Verona integron metallo-beta-lactamase (VIM) and Imipenemase (IMP) carbapenemases on cultured CRE isolates.

### 5.3. Study Procedures

#### 5.3.1. Meropenem, Tigecycline and Fosfomycin MIC Determination

**Figure 3:**
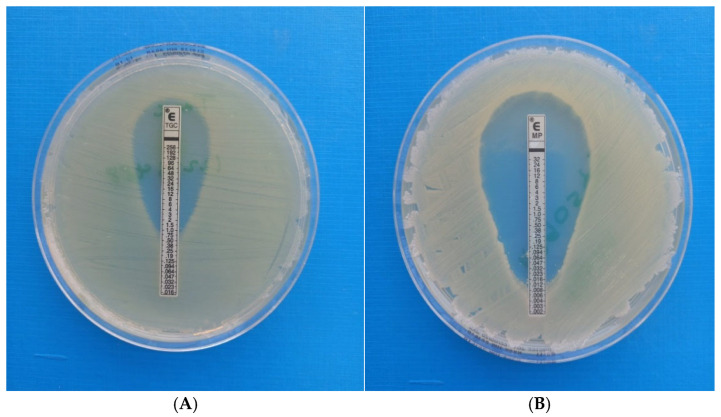
(**A**) E-test MIC testing for tigecycline; (**B**) E-test MIC testing for meropenem.

Meropenem (0.002–32 µg/mL), tigecycline (0.032–256 µg/mL) and fosfomycin (0.32–512 µg/mL). MICs were determined using the E-test (bioMérieux, France) method (Figure 3A,B). Mueller-Hinton agar plates (Thermo Fisher Scientific, Johannesburg, South Africa) were inoculated with a 0.5 McFarland bacterial suspension and incubated at 37 °C for 16–18 h in ambient air. The MIC was read at the concentration where the ellipse zone intercepted the strip. The interpretation of meropenem was according to CLSI M100 [14]. Tigecycline MICs were interpreted according to the Food and Drug Association (FDA) as there are no CLSI and EUCAST clinical breakpoints. Fosfomycin MICs could not be interpreted as clinical breakpoints do not exist [15].

#### 5.3.2. Colistin Broth Microdilution (BMD) Determination of MIC

**Figure 4:**
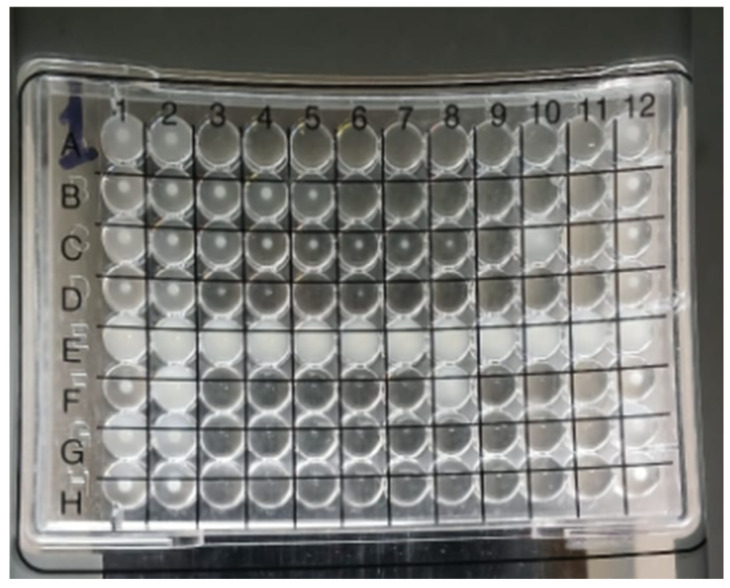
Sensititre colistin broth microdilution panel.

Colistin BMD verification using the Sensititre^®^ plate (Trek Diagnostic Systems, Cleveland, OH, USA) was performed at the microbiology lab using well-characterized clinical strains of both colistin resistant and susceptible CRE and XDR *Acinetobacter* spp. isolates and included American Culture Type Collection (ATCC) quality control strains (Figure 4). Colistin BMD has since become the recommended on-site AST methodology for colistin MIC determination with clinical breakpoint interpretation done according to CLSI [14,31].

#### 5.3.3. E-Test Synergy Testing (Fixed-Ratio) Methodology (AB Biodisk, Solna, Sweden)

**Figure 5:**
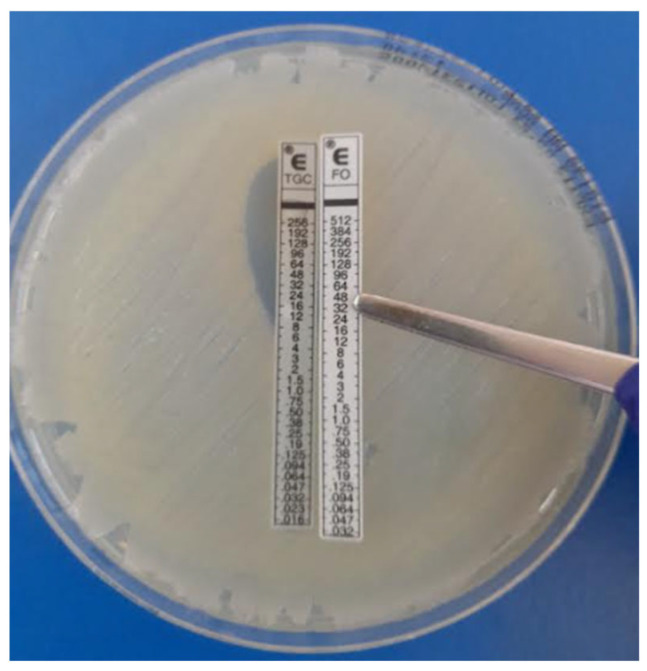
Tigecycline and fosfomycin combination—E-test synergy testing.

A total of 68 MDR GNB were tested for in vitro synergy comprising 38 CRE (*K. pneumoniae* (*n* = 35) and *E. coli* (*n* = 3)) and 30 XDR *Acinetobacter* spp. cultured isolates. Prior to ST, individual organism MICs for all isolates were determined for colistin, meropenem, fosfomycin and tigecycline as described above. The following E-test strips were used for the ST: colistin (0.016–256 µg/mL), meropenem (0.002–32 µg/mL), tigecycline (0.032–25 µg/mL) and fosfomycin (0.32–512 µg/mL). The antibiotic combinations tested per study isolate: colistin and meropenem, colistin and fosfomycin, colistin and tigecycline, meropenem and fosfomycin, meropenem and tigecycline and fosfomycin and tigecycline (Figure 5).

Strip A was placed on the inoculated Mueller Hinton (MH) agar plate surface that had been previously lawned with 0.5 McFarland of the test organism and left at room temperature for 60 min. The MIC position was then marked on the back of the agar plate, and strip A was then removed, cleaned with alcohol, and saved for reading results. Strip B was placed on the imprint of strip A and transposed vertically so that MIC A and MIC B would overlap in the same position. The agar plate with strip B was then incubated overnight at 35 °C for 24 to 48 h. The respective MIC strips were then used to read the combination MIC AB. The FIC index was calculated, and antimicrobial synergy of combination agents was noted, documented and interpreted accordingly (Table 3).

## 6. Conclusions

Therapeutic options for MDR GN pathogens have become a growing concern in the public healthcare sector. The currently prescribed combination of colistin and meropenem in the paediatric units at our local hospitals have been shown to have poor in vitro synergy against CRE and XDR *Acinetobacter* spp. pathogens. It must be emphasized that evaluating the efficacy of these antibiotic combinations by in vitro testing is essential to guide in vivo treatment. Tigecycline and meropenem featured the most synergistic combination amongst both CRE and XDR *Acinetobacter* spp. making it a potential therapeutic regimen in the paediatric unit. ST would be a valued addition to the local Microbiology laboratory testing repertoire facilitating individualized treatment options in patients with GN MDR bloodstream infections. Future clinical studies are recommended to gauge outcomes thereby amending treatment protocols accordingly in the management of such infections.

## Figures and Tables

**Table 1:** Antimicrobial MIC (µg/mL) distribution for test isolates by E-test and colistin BMD for CRE.

Carbapenem-Resistant *Klebsiella pneumoniae* (*n* = 35)										
Antimicrobial MIC (µg/mL)			≤1	2	4	6	8	16	24	32
Meropenem			8	2	3	2	2	6	0	12
Tigecycline			2	29	2	1	1	0	0	0
Colistin			32	3	0	0	0	0	0	0

**Antimicrobial MIC (µg/m** **L** **)**	**≤8**	**16**	**32**	**64**	**96**	**128**	**192**	**256**	**384**	**512**
Fosfomycin	5	3	8	4	2	4	2	1	0	6
**Carbapenem-** **R** **esistant *E. coli* (*n* = 3)**										
**Antimicrobial MIC (µg/m** **L** **)**			**≤1**	**2**	**4**	**6**	**8**	**16**	**24**	**32**
Meropenem			1	1	0	0	0	0	0	1
Tigecycline			1	2	0	0	0	0	0	0
Colistin			2	1	0	0	0	0	0	0

**Antimicrobial MIC (µg/m** **L** **)**	**≤8**	**16**	**32**	**64**	**96**	**128**	**192**	**256**	**384**	**512**
Fosfomycin	0	1	2	0	0	0	0	0	0	0

Meropenem MIC ≤ 1 µg/mL = susceptible; Tigecycline MIC ≤ 2 µg/mL = susceptible; Fosfomycin: no clinical susceptible breakpoints; Colistin: no clinical susceptible breakpoint.

**Table 2:** Antimicrobial MIC (µg/mL) distribution for test isolates by E-test and colistin BMD for XDR *Acinetobacter* species.

XDR *Acinetobacter* Species (*n* = 30)										
Antimicrobial MIC (µg/mL)			≤1	2	4	6	8	16	24	32
Meropenem			0	0	0	0	0	0	0	30
Tigecycline			2	27	1	0	0	0	0	0
Colistin			25	5	0	0	0	0	0	0

**Antimicrobial MIC (µg/m** **L** **)**	**≤8**	**16**	**32**	**64**	**96**	**128**	**192**	**256**	**384**	**512**
Fosfomycin	1	1	2	1	1	0	1	1	0	22

Meropenem MIC ≤ 2 µg/mL = susceptible; Tigecycline MIC ≤ 2 µg/mL = susceptible; Fosfomycin: no clinical susceptible breakpoints; Colistin: no clinical susceptible breakpoint.

**Table 3:** Fractional inhibitory concentration index interpretation for synergy testing [7].

Interpretation of FIC Index = MICAB/MICA + MICBA/MICB		Definition
Synergy	≤0.5	Describes the combination of antibiotics that produces an effect more potent than the combined potencies of each antibiotic.
Additive	>0.5 and ≤1.0	Denotes the effect of the drug combination being equal to the sum of the effects of each drug
Indifference	>1 and ≤4.0	Expressed when the drug combination is equal to the effect of the most active drug
Antagonism	>4.0	Describes the potency of the combination is less than the combined potencies of each antibiotic

## Data Availability

De-identified testing data will be available for review.

## Abbreviations

The following abbreviations are used in this manuscript:

multidrug-resistant	MDR
Gram-negative	GN
Gram-negative bacteria	GNB
species	spp.
antimicrobial resistance	AMR
carbapenem-resistant organisms	CROs
time-kill assays	TKA
Synergy testing	ST
Checkerboard Assays	CA
Epsilometer test	E-test
fractional inhibitory concentration	FIC
carbapenem-resistant Enterobacterales	CRE
extremely drug-resistant	XDR
low and middle-income countries	LMIC
bloodstream	BS
Bloodstream infections	BSI
Chris Hani Baragwanath Academic Hospital	CHBAH
National Health Laboratory Service	NHLS
standard operating procedures	SOPs
Antimicrobial susceptibility testing	AST
Clinical Laboratory Standards Institute	CLSI
Kirby Bauer disk diffusion	KBDD
oxacillinase	OXA-48
*Klebsiella pneumoniae* carbapenemase	KPC
New Delhi metallo-beta-lactamase	NDM
Verona integron metallo-beta-lactamase	VIM
Imipenemase	IMP
Minimum inhibitory concentrations	MICs
Food and Drug Association	FDA
European Committee on Antimicrobial Susceptibility Testing	EUCAST
Broth microdilution	BMD
Mueller Hinton	MH
